# Analysis of transcriptomic data sets supports the role of IL-6 in NETosis and immunothrombosis in severe COVID-19

**DOI:** 10.1186/s12863-021-01001-1

**Published:** 2021-11-14

**Authors:** Samanwoy Mukhopadhyay, Subrata Sinha, Saroj Kant Mohapatra

**Affiliations:** 1 National Institute of Biomedical Genomics, P.O. N.S.S, Kalyani, 741251 India; 2 Department of Biochemistry, All India Institute of Medical Sciences, New Delhi, 110029 India

**Keywords:** COVID-19, *IL-6*, Neutrophil extracellular trap, NETosis, Complement, Coagulation, Transcriptomics

## Abstract

**Background:**

There is an urgent need to understand the key events driving pathogenesis of severe COVID-19 disease, so that precise treatment can be instituted. In this respect NETosis is gaining increased attention in the scientific community, as an important pathological process contributing to mortality. We sought to test if indeed there exists robust evidence of NETosis in multiple transcriptomic data sets from human subjects with severe COVID-19 disease. Gene set enrichment analysis was performed to test for up-regulation of gene set functional in NETosis in the blood of patients with COVID-19 illness.

**Results:**

Blood gene expression functional in NETosis increased with severity of illness, showed negative correlation with blood oxygen saturation, and was validated in the lung of COVID-19 non-survivors. Temporal expression of *IL-6* was compared between severe and moderate illness with COVID-19. Unsupervised clustering was performed to reveal co-expression of *IL-6* with complement genes. In severe COVID-19 illness, there is transcriptional evidence of activation of NETosis, complement and coagulation cascade, and negative correlation between NETosis and respiratory function (oxygen saturation). An early spike in *IL-6* is observed in severe COVID-19 illness that is correlated with complement activation.

**Conclusions:**

Based on the transcriptional dynamics of *IL-6* expression and its downstream effect on complement activation, we constructed a model that links early spike in *IL-6* level with persistent and self-perpetuating complement activation, NETosis, immunothrombosis and respiratory dysfunction. Our model supports the early initiation of anti-*IL6* therapy in severe COVID-19 disease before the life-threatening complications of the disease can perpetuate themselves autonomously.

**Supplementary Information:**

The online version contains supplementary material available at 10.1186/s12863-021-01001-1.

## Background

COVID-19 disease caused by SARS-CoV-2 has rapidly become a center of intense scientific investigation, with emphasis on unravelling the biology for actionable knowledge. While the majority of the infected subjects are asymptomatic or mildly ill, a small percentage are severely ill with respiratory distress [1]. However, at present, it is difficult to predict with certainty the patients at high-risk for clinical severity and poor outcome, although multiple pathophysiological processes have been proposed, such as, cytokine storm [2, 3], coagulation and complement activation [4], neutrophil extracellular trap - NETosis [5]. These studies have also led to predictive biomarkers, such as, neutrophil to lymphocyte ratio [6, 7] and interleukin 6 (*IL-6*) expression [2, 4].

Insight into cytokine dysregulation has driven therapeutic advances, such as, anti-cytokine tocilizumab (*IL-6* receptor antagonist) for severely ill patients of COVID-19 [8, 9]. Such treatment is premised on the induction of intra-pulmonary inflammation by SARS-Cov-2 infection that ultimately leads to severe local vascular dysfunction including micro-thrombosis, haemorrhage and pulmonary intravascular coagulopathy [10]. It has been suggested to start tocilizumab early, in order to avoid mechanical ventilation [11], although the best timing for the treatment is still being investigated [12]. Therefore, it is important to understand the temporal and/or causal relationship of the cytokine up-regulation with coagulopathy and respiratory dysfunction.

The prothrombotic state (contributing to pulmonary dysfunction in COVID-19) is explained in terms of Neutrophil extracellular traps (NETs) that originate from decondensed chromatin of neutrophils that can trigger immunothrombosis. Critically ill patients of COVID-19 show significantly higher plasma levels of MPO-DNA complex, a marker of NETosis. Factors triggering NETs were significantly increased in COVID-19 and pulmonary autopsies confirmed NET-containing microthrombi with neutrophil-platelet infiltration. The authors concluded that NETs triggering immunothrombosis may partly explain the prothrombotic clinical presentations in COVID-19 [5].

In view of the pivotal role of cytokines (especially *IL-6*) and NETosis in biology of COVID-19 host response, we performed a deep and focussed investigation into *IL-6,* NETosis, complement and coagulation in published data from multiple patients of COVID-19 with varying illness severity. The primary goal was to dissect the transcriptomic dynamics of the functional modules and examine if there is a therapeutic window for drugs targeting specific pathophysiological mechanisms, such as *IL-6* blockade or inhibition of NETosis.

## Results

Human transcriptomic data were extracted from published data sets of patients of COVID-19 from different tissues: whole blood (longitudinal sampling) [13], peripheral blood mononuclear cells (PBMC) [14], and lung tissue [15]. Whole blood was especially selected because it includes neutrophils that are directly responsible for formation of NETs.

### Up-regulation of NETosis in COVID-19

**Fig. 1:**
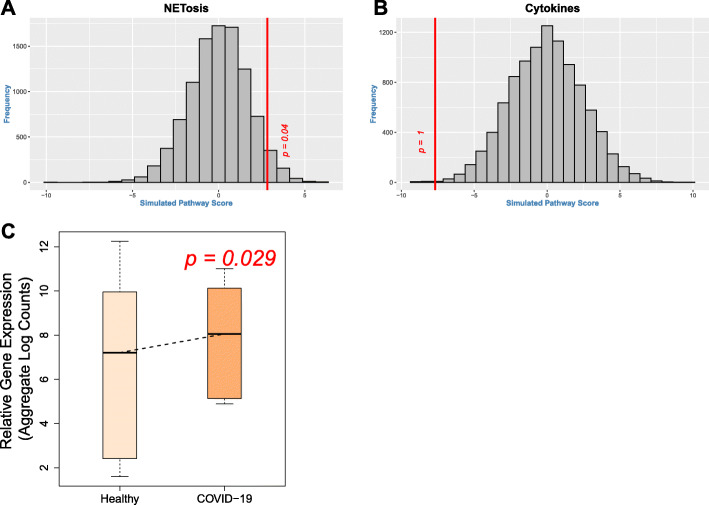
Up-regulation of genes functional in NETosis in the blood of patients with COVID-19. (**A**) For the gene set functional in NETosis, pathway score (red vertical line) was calculated by weighted averaging of t-statistic between control group (10 healthy subjects, single time point) and COVID-19 group (3 cases, multiple time-points). The histogram (gray bars) represents the null distribution of the pathway score calculated for each of the 10,000 iterations of permuting the sample labels. The position of the red vertical line (observed pathway score) with respect to the histogram (null distribution) suggests that the pathway is significantly up-regulated in the blood of COVID-19 patients. As shown, there is significant (*p* = 0.04) up-regulation of the gene set functional in NETosis. (**B**) Calculation of the pathway score and the null-distribution of the cytokine gene set has been performed as mentioned for NETosis. As shown, there is no (*p* = 1) up-regulation of cytokine gene set in COVID-19. (**C**) For validation of NETosis, gene expression data of healthy control (*n* = 6) and COVID-19 (*n* = 7) were extracted from an independent cohort [14]. Box plot shows up-regulation of NETosis genes in COVID-19 cases compared to the control group. Gene expression data were extracted from the data set E-MTAB-8871 [13] for panels A and B; and from the data set GSE150728 [14] for panel C

Targeted analysis was performed to study the extent of differential expression of two gene sets in whole blood: cytokine genes and genes functional in NETosis. Gene set functional in NETosis was significantly up-regulated in whole blood of patients of COVID-19 [13]. Gene set enrichment analysis (permutation testing) revealed that the genes functional in NETosis were strongly up-regulated in the blood of COVID-19 patients compared to healthy subjects (Fig. 1A). On the other hand, there was no evidence of broad up-regulation of cytokine gene set in the blood of the patients (Fig. 1B). Up-regulation of genes functional in NETosis was validated in an independent data set of COVID-19 (Fig. 1C).

### Association of NETosis up-regulation with disease severity

**Fig. 2:**
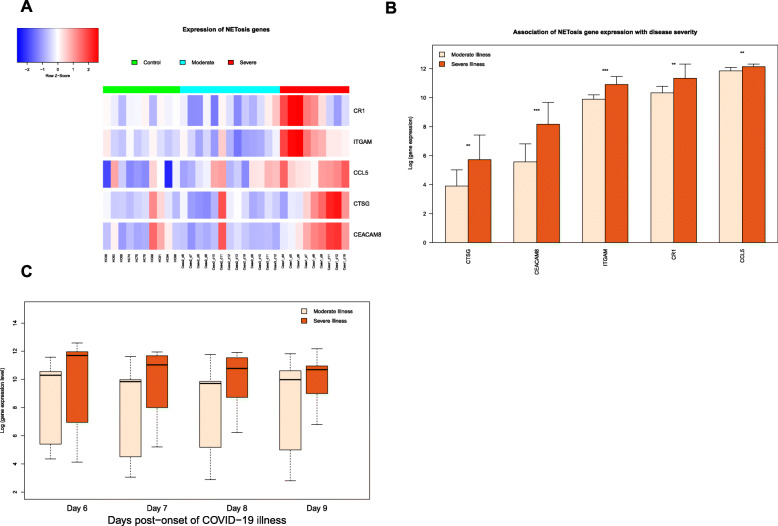
Expression of genes functional in NETosis increases in blood with COVID-19 disease severity. Gene expression data [13] were collected from healthy control subjects (*n* = 10), and COVID-19 patients sampled at multiple time points: moderate illness (2 patients, 13 samples), severe illness (1 patient, 9 samples). (**A**) Column sidebar on the top marks healthy control as green, moderate illness as cyan, and severe illness as red. Each row represents a gene participating in NETosis. The colour of the cell represents the level of expression, low as blue and high as red. Magnitude of up-regulation of NETosis genes is greater in the case with severe COVID-19 illness (as shown by the greater number of red cells for case 1 – severe illness). (**B**) Each bar represents the mean log-expression of a gene across all-time samples from either moderate or severe illness. Error bar represents standard deviation of gene expression levels across all samples in that group of illness severity. For most of the genes there is a higher level of gene expression in severe illness compared to moderate illness. Significance of up-regulation in severe illness was assessed by t-test and is indicated with an asterisk over the bars (** *p* < 0.01; *** *p* < 0.001). (**C**) For each of the days 6, 7, 8 and 9 (post-onset), box plot shows higher level of gene expression functional in NETosis in a patient with severe COVID-19 illness compared to a patient with moderate COVID-19 illness

As the heat map (Fig. 2A) shows the magnitude of up-regulation is greater in the severe COVID-19 illness (Case 1) compared to cases with moderate illness (Cases 2 and 3). In general, more genes functional in NETosis are up-regulated (red cells) in severe illness compared to moderate illness. The genes shown in Fig. 2A are shown in the bar graphs in Fig. 2B. Average level of expression of each gene across all time points was compared between moderate illness (pink bar) and severe illness (orange bar). For each of these genes, level of expression is higher (and for many of these genes, the difference is statistically significant) in severe illness compared to moderate illness (Fig. 2B).

Further, level of gene expression was compared between severe and moderate illness for fixed time points (days post-onset of illness). For each of the four time points (Day 6, 7, 8 and 9 post-onset, where such paired data were available), box plot was drawn to show up-regulation of NETosis in severe illness compared to moderate illness (Fig. 2C). There is an upward trend in gene expression from moderate illness to severe illness at each day post-onset, suggesting sustained up-regulation of genes functional in NETosis in severe illness.

### Up-regulation of genes functional in NETosis in lung of COVID-19 patients

**Fig. 3:**
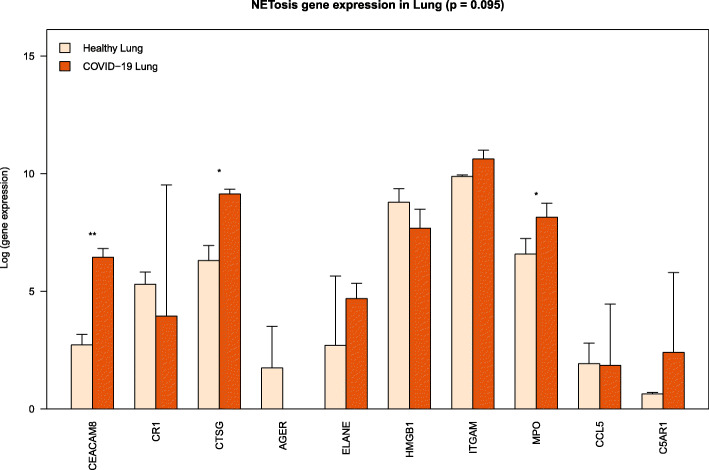
Expression of genes functional in NETosis increases in lung with COVID-19 disease severity. Each bar represents the average log-gene expression in the lung of COVID-19 non-survivors (COVID-19 Lung; *n* = 2) and post-mortem lung tissue of uninfected individuals (Healthy Lung; n = 2) [15]. Error bar represents standard deviation of log-gene expression. Significance of up-regulation in severe illness was assessed by t-test and is indicated with an asterisk over the bars (* *p* < 0.1; ** *p* < 0.01). The genes functional in NETosis are up-regulated in the lungs of the patients with COVID-19

NETosis up-regulation was observed in lung tissue from deceased COVID-19 patients. Genes functional in NETosis are up-regulated in the lungs of COVID-19 patients compared to healthy control (Fig. 3). These genes include cathepsin G, CEA cell adhesion molecule 8, complement *C3b/C4b* receptor 1, integrin subunit alpha, C-C motif chemokine ligand 5.

### Up-regulation of NETosis is associated with higher neutrophil to lymphocyte ratio (NLR)

**Fig. 4:**
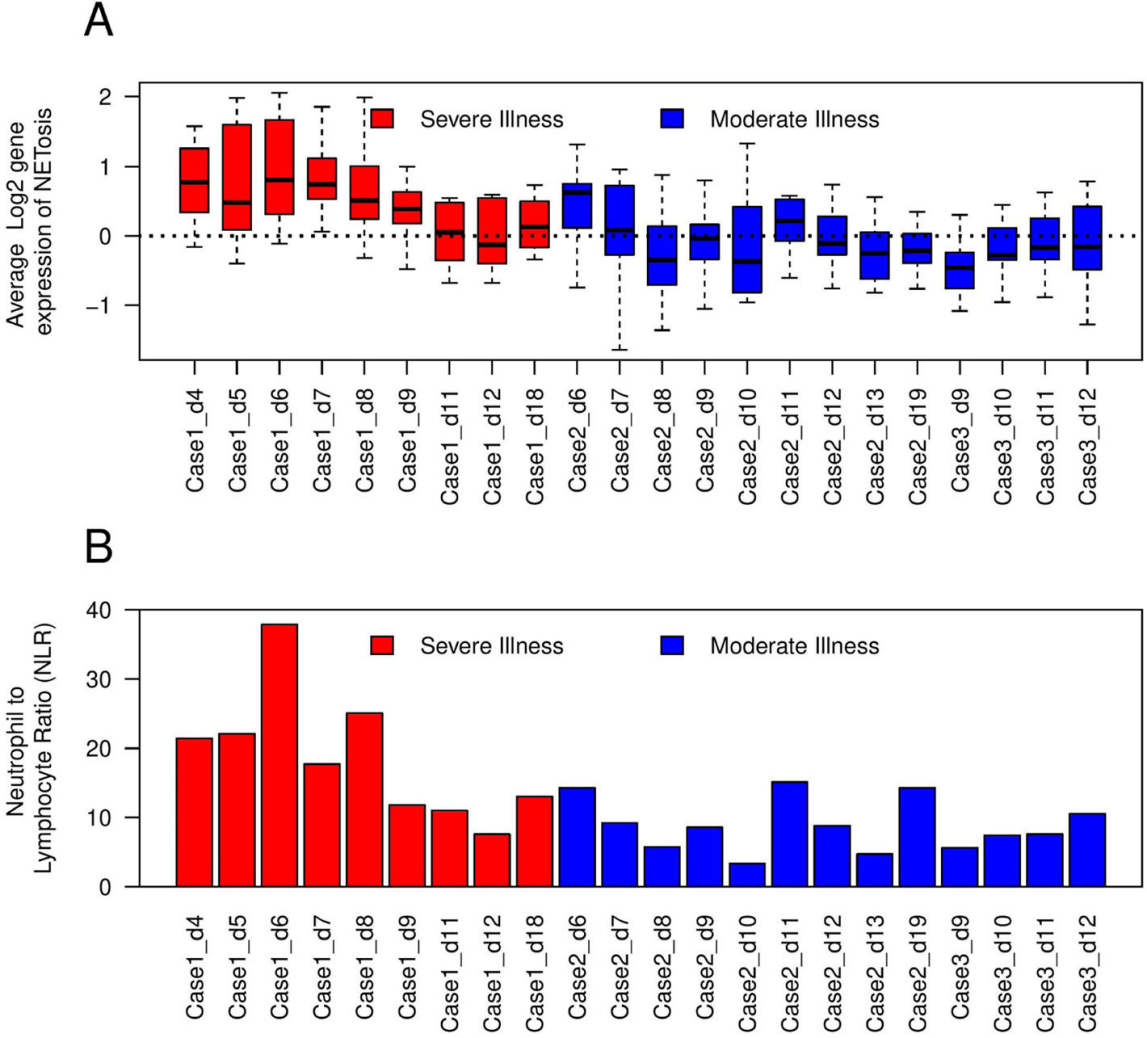
Expression of genes functional in NETosis is positively correlated with Neutrophil to Lymphocyte ratio in COVID-19. Gene expression data were extracted from [13]. (**A**) Each box represents a single sample (color-coded red for the patient with severe illness and blue for the patients with moderate illness). Dotted line represents baseline expression (average gene expression in healthy subjects). The samples from the severe case, especially the early time points, have higher expression (up-regulation) of NETosis genes compared to the cases with moderate illness. With passing days from the onset of COVID-19, gene expression tends to return to baseline. (**B**) Each bar represents neutrophil to lymphocyte ratio (NLR) in peripheral blood in that sample. NLR is increased at the early time points in severe illness. With passing days from the onset of COVID-19, NLR is reduced. Generally, there is agreement in the trend of NETosis gene expression and NLR in the patients of COVID-19 (correlation coefficient = 0.8)

NETosis up-regulation was associated with increased NLR in the blood. As shown in Fig. 4A, there is up-regulation of genes functional in NETosis at a level higher in the severe case (red) compared to the moderate cases (blue). Additionally, NLR is higher in the blood of severe case compared to moderate cases. With time, both NLR and NETosis gene expression return to baseline in the severe case (red boxes and red bars respectively for case 1 in Fig. 4A and B). NLR is positively correlated with gene expression functional in NETosis.

### Negative association between NETosis and respiratory function

**Fig. 5:**
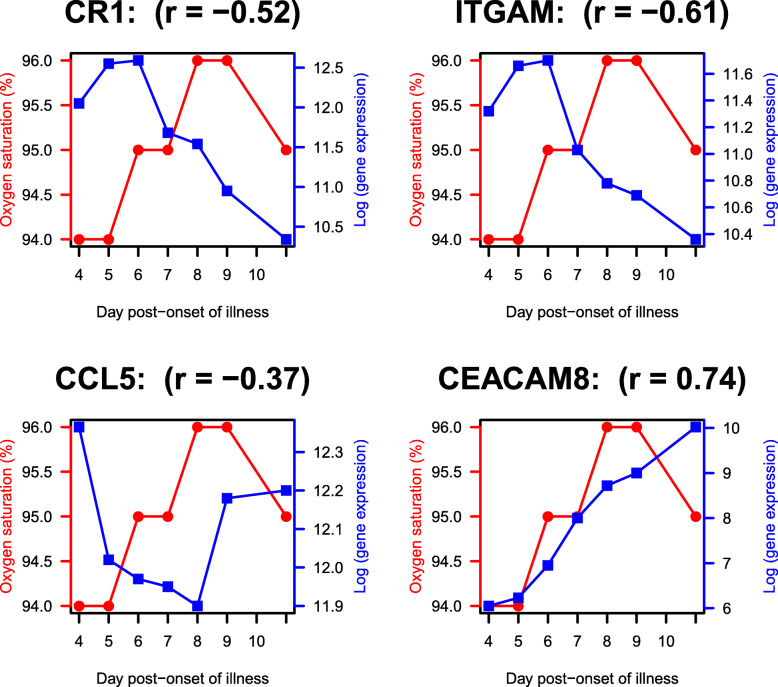
Gene expression functional in NETosis is negatively correlated with oxygen saturation in severe COVID-19 illness. Temporal line plots of selected NETosis genes in the severe patient (Case 1) over days of illness. Each plot corresponds to one gene, with the red line representing oxygen saturation (%), and the blue line representing level of gene expression. Pearson correlation coefficient (r) is negative between oxygen saturation and level of expression of these genes. In contrast to other genes shown here, CEACAM8 up-regulation is sustained over time. Gene expression data were extracted from [13]

In the severely ill patient of COVID-19, line plots were drawn to show the reciprocal relationship of gene expression with oxygen saturation (%). As shown in Fig. 5, there is up-regulation of genes (functional in NETosis) at the time of low oxygen saturation and down-regulation otherwise. This is also proven by negative correlation coefficient for the genes (CR1: -0.52, CCL5: -0.37, ITGAM: − 0.61).

### Up-regulation of complement pathway

**Fig. 6:**
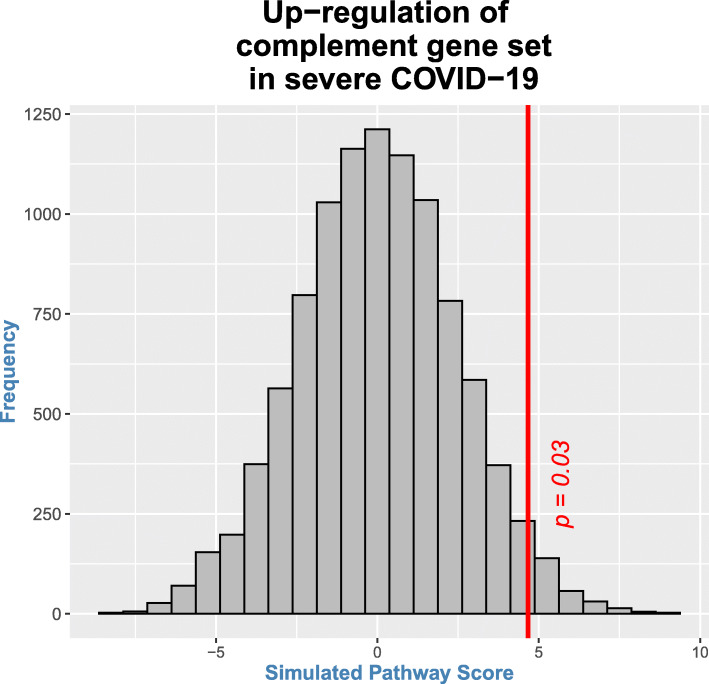
Gene expression of Complement pathway is significantly up-regulated in severe COVID-19 illness. The histogram (grey bars) represents the null distribution of pathway score calculated by weighted averaging of individual relative expression levels (in severe illness with respect to that in moderate illness) for genes functional in the complement pathway. Only those genes of the pathway present on this platform [13] were included in the analysis. The red vertical line represents the observed pathway score for each set. The position of the red line with respect to the histogram (i.e., toward the right tail) suggests that there is significant up-regulation of the gene set functional in complement pathway (*p* = 0.03) in severe COVID-19 illness

**Fig. 7:**
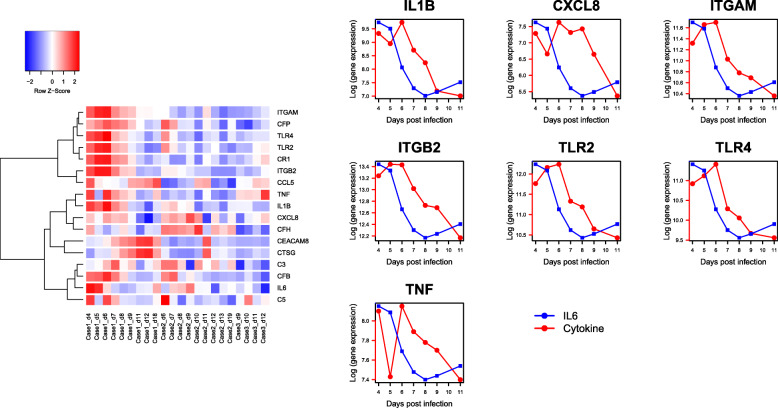
KEGG pathway Complement and Coagulation cascades – *Homo sapiens* (human) (hsa04610) [16] has been coloured on the basis of differentially expressed genes in COVID-19 using data from [4]. The up-regulated genes are shown in red. The down-regulated genes are shown in green. As displayed, multiple genes of the complement and coagulation cascades are up-regulated in COVID-19

Genes belonging to the complement pathway were extracted and subjected to gene set enrichment analysis (permutation testing). As shown in Fig. 6, there is significant up-regulation of the genes functional in the complement pathway. Additionally, multiple genes functional in the coagulation cascade are also observed to be up-regulated (Fig. 7).

### IL-6 segregates with complements

**Fig. 8:**
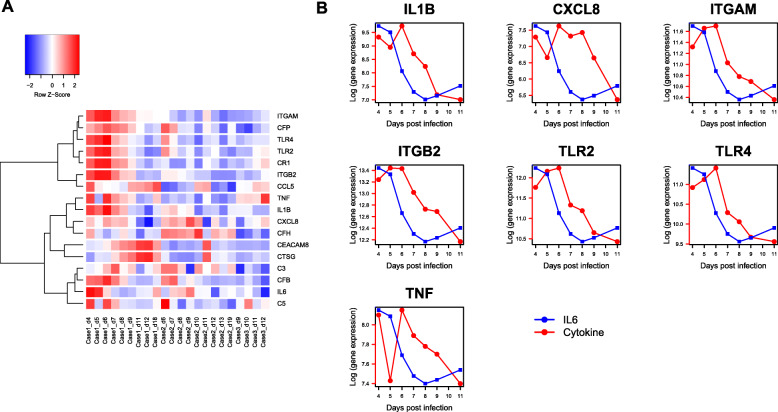
(**A**) Unsupervised clustering reveals segregation of IL-6 with complement genes. The heat map was generated from all-time samples of the 3 cases of COVID-19 (Case 1 - severe illness, Cases 2, 3 - moderate illness) and log (gene expression) of four cytokine genes (IL-6, IL-8, TNFα, IL1β) and genes functional in NETosis (including complement factors) [13]. Unsupervised hierarchical clustering reveals segregation of IL-6 with the complement factors C3, C5 and CFB while the other 3 cytokines (IL-8, TNFα, IL1β) were assigned to a separate clade. The samples are arranged according to the case IDs (from left to right: Case 1, Case 2, Case 3), with increasing days post-onset of illness from left to right. Gene expression data were extracted from [13]. (**B**) Temporal Line plot IL6 vs NETosis genes in severe COVID-19. These line plots show the temporal relation of other important cytokines along with IL-6

We performed an unsupervised clustering of four cytokines *(IL-6, IL-8, TNFα, IL1β)* with genes functional in NETosis (including complement genes). Gene expression data (log-scale) of all-time samples from the three patients of COVID-19 were used. Hierarchical clustering revealed segregation of *IL-6* with complement factors *C3, C5* and CFB (Fig. 8). The other 3 cytokines were assigned to a separate clade. Since the data are captured from peripheral blood of the patients during the time-course of illness, this result suggests a possible role of *IL-6* in the gene expression dynamics of complement genes.

### Changing expression level of IL-6

**Fig. 9:**
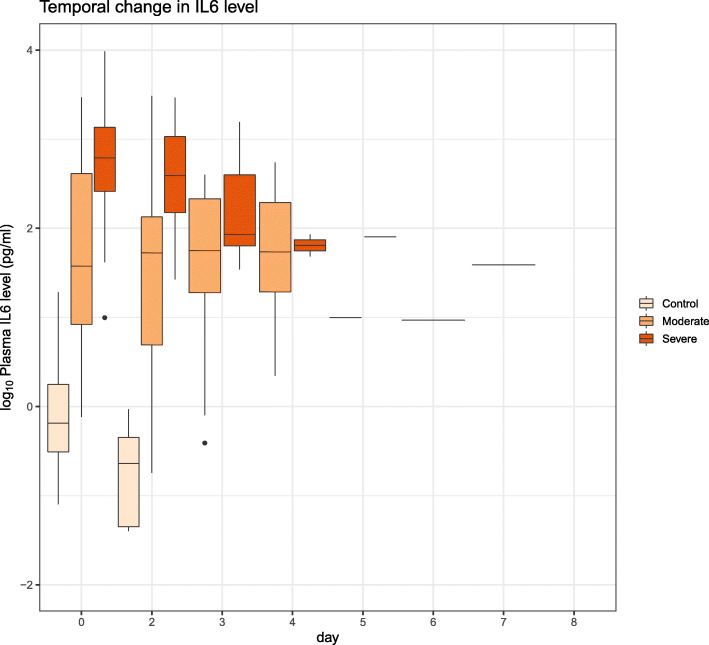
Temporal change in IL-6 expression differs between severe and moderate COVID-19 disease. Box plot shows temporal change in plasma IL-6 level in control and patients with (moderate and severe) COVID-19 illness. At the early time points, IL-6 level is higher in the cases with severe illness compared to the cases with moderate illness. At the later time points, the level of IL-6 approaches that in the cases with moderate illness. Data were extracted from [17]

**Fig. 10:**
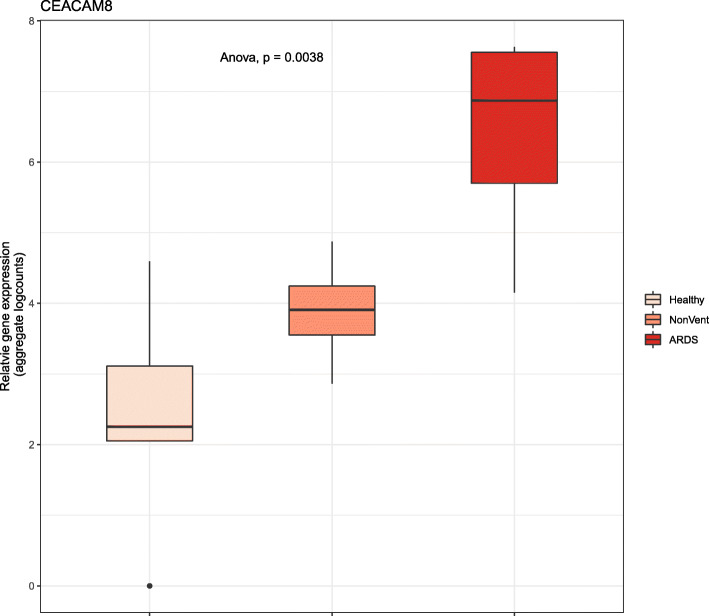
CEACAM8 gene expression increases with COVID-19 disease severity. CEACAM8 gene expression in PBMC of healthy control (*n* = 6) and COVID-19 (*n* = 7) were extracted from published data set [14]. Group-level expression (aggregate log counts) data are shown in the box plot, with monotonic up-regulation of CEACAM8 genes from the control group, to COVID-19 cases without ARDS (NonVent) and with ARDS. CEACAM8 up-regulation is a signature of immature or developing neutrophil, a neutrophil subtype associated with COVID-19. Statistical significance of the difference in DNASE1 transcript levels was calculated using analysis of variance (Anova)

Box plot of longitudinal *IL-6* profiling in three groups of subjects (healthy control, moderate illness, severe illness) revealed that the magnitude of up-regulation is greatest early in the disease process (Fig. 9). With increasing days post-onset, the level of expression in the severe illness approaches that in the moderate illness (Fig. 9). The reduction in *IL-6* expression coincides with an increase in the expression of genes functional in NETosis, such as *CTSG* and *CEACAM8* (Fig. 10).

### Reduction in the level of DNASE1 expression

**Fig. 11:**
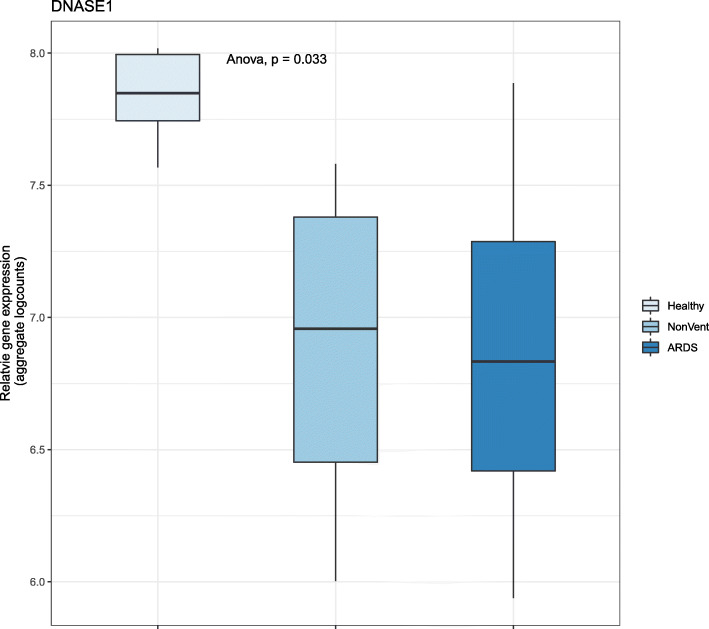
DNASE1 gene expression decreases with COVID-19 disease severity. DNASE1 gene expression in PBMC of healthy control (n = 6) and COVID-19 (n = 7) were extracted from the published data set [14]. Box plot of group-level gene expression (aggregate log counts) of DNASE1 shows monotonic down-regulation (with increasing severity of illness) from the control group, to COVID-19 cases without ARDS (NonVent) and with ARDS. Statistical significance of the difference in DNASE1 transcript levels was calculated using analysis of variance (Anova)

In view of the role of *DNASE1* in clearance of NETs, we explored the level of its expression in the patients of COVID-19 with varying illness severity. As shown in Fig. 11, there is significant down-regulation of *DNASE1* in the COVID-19 patients, with greater down-regulation in the COVID-19 patients with ARDS.

### A model connecting the rise in IL-6 level with NETosis leading to immunothrombosis and ARDS

Considering the robust up-regulation of NETosis, complement pathways and transient early rise of *IL-6* in severe cases of COVID-19, a model is proposed. *IL-6* is a likely trigger for complement up-regulation and NETosis that leads to coagulation, platelet activation and positive feedback on neutrophil activation. The model is based on the temporal dynamics of *IL-6* and co-expression with complement genes *C3* and *C5,* activation of neutrophil, formation of neutrophil extracellular trap, activation of platelets, coagulation, and positive feedback from platelet to neutrophil. Post-*IL-6* spike, a vicious cycle of NETosis and thrombosis ensues and sustains illness severity and ARDS.

## Discussion

Unbiased analysis of transcriptome data reveals that gene set functional in NETosis is strongly up-regulated in the blood of COVID-19 patients. The up-regulation is statistically significant and is higher in magnitude in severe illness than in moderate illness. Paired testing reveals that from day 6 until day 9, there is significant and sustained elevation of gene expression functional in NETosis in severe illness compared to moderate illness. Of note, death of the patients of COVID-19 occurs primarily due to the complications arising from SARS-CoV-2-associated acute respiratory distress syndrome. NETosis is known to cause immunothrombosis and respiratory dysfunction in COVID-19 [5]. We present transcriptional evidence of increased NETosis in both peripheral blood and lung tissue of COVID-19 patients. Additionally, time-course expression data from a case of severe COVID-19 reveal negative association of NETosis with respiratory function (oxygen saturation). Together, these findings are consistent with NETosis as an underlying mechanism for a prothrombotic state in blood leading to respiratory dysfunction.

Transcriptional profiling of nasopharyngeal swabs from COVID-19 patients have demonstrated upregulation of complement and coagulation pathway associated with mortality and morbidity [4]. Our analysis (of data from [13]) also revealed significant up-regulation of the complement pathway genes in severe COVID-19 illness. NETs act as scaffolds for both coagulation and complement activation, and the three pathways (NETosis, complement and coagulation) are considered a single coordinated biological process [18]. NETosing neutrophils have been shown to activate complement via alternative and non-alternative pathways [19]. Also, activated macrophages are known to cause induction of complement factors. The supernatant of macrophage that causes overexpression of the complement factors C3 and CFB are enriched in IL-6 [20], which is consistent with our observation of segregation of complement factors *C3* and *CFB* with *IL-6* but not with the other pro-inflammatory cytokines (*IL-8, IL-1β* and *TNFα*) in COVID-19 illness. While cooperation among different components of NETosis-complement-coagulation consortium protects the host against both haemorrhage and infection [18], unchecked NETosis causes immunothrombosis and leads to acute respiratory distress in COVID-19 illness [5].

Plasma from both COVID-19 patients [5] and patients of sickle cell disease (SCD) with vaso-occlusive crisis (but not from steady state plasma of SCD) cause significant increase in NETosis [21]. The level of *IL-6* is observed to be high in the plasma from these patients. Up-regulation of *IL-6* signaling has been observed in nasopharyngeal swab [4], lung [15] and has been associated with poor outcome of COVID-19 [2, 4, 15]. Interestingly, Mann and colleagues [7] observed an early rise of *IL-6* level in critically ill patients of COVID-19, which progressively decreased over time even if the patient did not survive. In data from a different cohort [17], we also observed an early spike of blood expression of *IL-6* in severe COVID-19 illness, which returns, over time, to levels comparable with moderate illness. Together these findings support a dynamic shift in *IL-6* level in severe illness – with potential mechanistic and therapeutic significance of *IL-6* in the early time window.

**Fig. 12:**
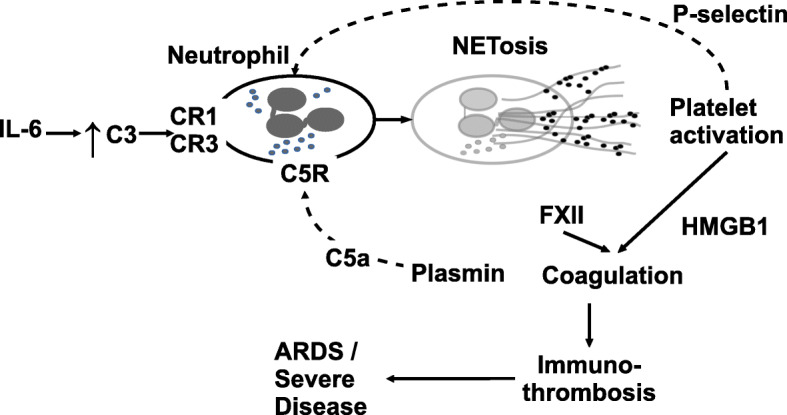
A simple model with IL-6, complement factors, NETosis, and Coagulation leading to immunothrombosis. IL-6 induces expression of C3 and CFB leading to complement activation. C3 engages complement receptors (CR1, CR3) on neutrophils and activates formation of NET [18]. The components of NET activate platelets which secrete HMGB1, inducing coagulation. Negatively charged NETs can bind and activate circulating glycoprotein FXII (a zymogen produced by liver) that induce coagulation. Plasmin cleaves C5 to active C5a which in turn activates Neutrophil by binding via C5R [18]. Activated platelets engage with activated neutrophils through binding of P-selectin to PSGL-1 [18]. NETosis, complement activation and coagulation are functionally interrelated and together produce immune-thrombosis. As the blood vessels in the lungs are clogged, it leads to acute respiratory distress and high mortality

It seems likely that the spike in *IL-6*, secreted by the macrophages responding to the viral entry, triggers NETosis in the patients with severe COVID-19, leading to a complex interaction among NETosis, complement and coagulation pathways [18], pulmonary immune thrombosis and acute respiratory distress. *IL-6* is known to stimulate thrombosis in platelet-dependent and platelet-independent manner [5, 22]. With time, while *IL-6* levels in patients with severe illness approach that of patients with moderate illness (Fig. 9), NETosis and complement activation are sustained. Therefore, inhibition of *IL-6* signaling is most beneficial before sustained up-regulation of NETosis by a positive feedback loop (Fig. 12). In the later phase, *IL-6* levels are similar in severe and moderate illness, but NETosis-complement-coagulation leads to immunothrombosis, compounded by reduced clearance of the NETs (due to decreased levels of DNASE1), ultimately leading to acute respiratory distress in the severe cases. In this phase, a different strategy is called for, such as, inhibition of complement and NETosis. Level of *IL-6* upon admission can be used as a prognostic marker of outcome [2] and for prioritization of anti-*IL-6* therapy.

Neutrophil to lymphocyte ratio (NLR) is proposed as a prognostic biomarker of disease severity and organ failure in COVID-19 [6]. In general, there is an increased number of neutrophils in blood, which, along with lymphopenia, contribute to high NLR. There is also increased neutrophil activation of genes functional in formation of NET. The host gene expression in COVID-19 disease is consistent with neutrophilia commonly observed in severe COVID-19 illness resulting in increased formation of Neutrophil Extracellular Traps (NETs). Therefore, NETosis adequately explains the prognostic power of NLR, and extends itself as a fundamental dysregulation underlying COVID-19 disease severity, respiratory distress and mortality.

While there is an increased number of neutrophils in the patients of COVID-19, it is not clear if these are the usual neutrophils of healthy blood. Wilk and colleagues [14] observed a novel kind of “developing neutrophil” in the blood of COVID-19 patients. These neutrophils express high levels of *CEACAM8*, a marker of immature neutrophils that are higher in men and pregnant women compared to non-pregnant women [23]. Notably, mortality and morbidity in COVID-19 has been consistently associated with gender of the patients, with male patients at a higher risk of poor outcome [3, 24]. The role of any hormonal influence on neutrophil type and activation (and NETosis) in COVID-19 outcome remains to be elucidated.

Sepsis appears as the single most frequent factor associated with mortality in COVID-19 [25]. Similar to COVID-19, sepsis is also associated with coagulopathy [26, 27]. It is likely that the dynamic cytokine (*IL-6*) dysregulation induces NETosis and coagulation in other non-COVID causes of sepsis. Thus, temporal and precise mechanistic therapy targeting *IL-6* and NETosis shall potentially benefit critically ill patients of both COVID-19 and sepsis.

Level of IL-6 gene expression is known to be associated with the − 174 G/C polymorphism (rs1800795) [28], and we considered the possibility that this variant is associated with severe COVID-19 and poor outcome. A search on the PubMed with the query string “rs1800795 and COVID-19” returned only two articles that tested for association between this polymorphism and IL-6 level, severity, and outcome in COVID-19 patients [29, 30]. In one study (*n* = 70) [29], with patients belonging to two groups, severe (high IL-6 and macrophage activation syndrome - MAS), and non-severe (no-MAS, low IL-6), G allele is significantly associated with MAS. In the other study (*n* = 71) [30], C allele is associated with higher mortality and IL-6 levels. These two studies show contradictory results: G allele appears to be the risk in one study but protective in the other. Therefore, it is difficult to come to any conclusion regarding the association of this polymorphism with COVID-19 outcome. On the other hand, an IL-6 spike can be caused by events such as viral load which can over-ride the effect (if any) of the host genetic variants. We have observed that the increased level of IL-6 in severe illness (compared to moderate illness) is most accentuated in the early stage of illness (Fig. 9). This can be explained by decreased viremia that no longer stimulate IL-6 release but cannot undo the (proposed) cascading effects on complements, NETosis and ultimately thrombosis.

## Conclusions

In conclusion, we present evidence from the analysis of several transcriptomic data sets of NETosis and complement activation in COVID-19, especially in severe disease. There is an early spike in *IL6* transcription in early infection, which is not so apparent in later stages. The early spike could trigger NETosis in severe cases. Over time, however, the level of *IL-6* expression fluctuates, while the patterns suggestive of NETosis and complement activation persist. This could indicate, as suggested by our model, that after initiation by *IL6*, NETosis and complement activation can continue autonomous to the initiating signal. This is a possible explanation to the variable results of anti-*IL6* therapy in severe COVID-19 disease. This also supports the early initiation of anti-*IL6* therapy before the life-threatening complications of the disease can perpetuate themselves autonomously.

## Methods

This study follows retrospective, case-control study design in order to prove that NETosis exists in COVID-19 cases. After this the study focuses on association of NETosis with disease severity. Then it associates the role of probable factors along with NETosis playing a role in disease severity.

### Selection and preprocessing of the data

Gene expression data of different covid-19 studies were downloaded from NCBI GEO [31], ArrayExpress [32] and other data repository portals (Table 1). The raw count matrix data were quantile normalised and log transformed, if necessary. The normalised data were then stored as individual “expressionSet” objects and subjected to downstream analysis. All analyses were performed in the R programming language [33].

**Table 1:** Study characteristics Table showing study characteristics with sources for data sets used in the current study

Database Link	Author	Article Title	DOI	Country	Tissue	Ref.	Used in the Figure of manuscript
https://covidgenes.weill.cornell.edu/	Ramlall	Immune complement and coagulation dysfunction in adverse outcomes of SARS-CoV-2 infection	10.1038/s41591-020-1021-2	USA	Nasopharyngeal Swabs	4	7
E-MTAB-8871 (ArrayExpress)	Ong	A Dynamic Immune Response Shapes COVID-19 Progression	10.1016/j.chom.2020.03.021	China	Whole blood	13	1, 2, 4–6, 8
GSE150728 (NCBI GEO)	Wilk	A single-cell atlas of the peripheral immune response in patients with severe COVID-19	10.1038/s41591-020-0944-y	USA	PBMC	15	1, 10, 11
GSE147507 (NCBI GEO)	Blanco-Melo	SARS-CoV-2 launches a unique transcriptional signature from in vitro, ex vivo, and in vivo systems	10.1101/2020.03.24.004655	USA	Primary cell cultured from Lung	14	3
https://www.immport.org/shared/home; study ID SDY1655	Lucas	Longitudinal analyses reveal immunological misfiring in severe COVID-19	10.1038/s41586-020-2588-y	USA	Serum	17	9

### Permutation based gene set enrichment analysis

Any pathway with 10 or less number of genes was discarded from analysis. For each gene, t-statistic was computed to denote change in gene expression in the case group compared to the control group. For each pathway, a score was calculated by weighted averaging (i.e., sum of the gene-level t-statistics divided by the square root of the number of genes in the pathway) of all gene-level t-statistics for the pathway. Significance of the observed pathway score was calculated by permutation testing performed in the following manner. In each permutation, the samples were randomly re-labelled as case and control, with calculation of a simulated pathway score. This was done 10,000 times generating 10,000 simulated values representing the null distribution of the pathway score. Deviation of the observed pathway score from the null distribution was quantified by the fraction of times that the simulated score was more extreme than the observed score. This result was assigned as a permutation *p*-value of the observed pathway score. Pathway enrichment analysis was performed using code modified from the R function gseattperm() of the package Category [34].

### Selection of cytokine and NETosis genes

The genes belonging to the two groups were selected from relevant literature describing cytokine storm [35] and NETosis [18]. The list of genes functional in NETosis is provided in Supplementary Table 1. (provided as an additional file)

### Method for deconvolution

We used the CIBERSORTx [36] for deconvolution of transcriptome data i.e., to find the cellular components in the sample through search for similarity of expression with reference expression values of specific cell types. A gene by sample expression matrix was created with the instructed format of the web portal guidelines. The reference immune cell gene expression was selected from the lymphocyte signature matrix (LM22) [37]. The analysis was run without batch correction (only one dataset at a time) and normalisation (as instructed for RNAseq data) with 1000 permutations. The resulting sample by immune cell-fraction matrix was downloaded in comma separated values (.csv) file format and analysed to estimate neutrophil to lymphocyte ratio (NLR).

## Supplementary Information

**Additional file 1.**

## Data Availability

Data and R code are available in a github repository named covid-19 (“https://github.com/skm-lab/covid-19”) for reproduction of all images and tables in the manuscript. **Processed data:** Any reader could find processed .rda files containing gene expression data within the folder named “Data”. **Raw data:** Table 1 contains the name of the article/study, the database on which the data is deposited, link of the particular study in the database. Any reader could find the raw data by navigating through Table 1.

## Abbreviations

NET: Neutrophil Extracellular Traps
NLR: Neutrophil to Lymphocyte Ratio
PBMC: Peripheral Blood Mononuclear Cells
ARDS: Acute Respiratory Distress Syndrome
NCBI: National Center for Biotechnology Information
GEO: Gene Expression Omnibus
